# Editorial

**Published:** 1897-12

**Authors:** 


### Editorial.

Dear Doctor:—As you no doubt are fully aware, there is a constant accumulation of evidence showing many nervous diseases and derangements are caused by eye-strain.

The increasing number of physicians who are availing themselves of my offer to send them a diagnosis of each case they send, shows me there is a growing desire among the fraternity to know the exact state of the eye while pursuing a medical course of treatment. If you have not already looked upon the eye as an important factor in causing headache and nervous trouble, you still may have some cases where you suspect that eye-strain is aggravating the trouble. If you desire, send them to me, and know for a certainty whether your suspicions are correct.

I have a special "dark-room" for retinoscopic and ophthalmoscopic examinations, and every other facility for doing thorough work. My method is to carefully diagnose each case for any errors of refraction or muscular insufficiency, and to report all particulars to the physician sending me the patient. There is no charge for the examination, and glasses only when necessary are recommended, which are prepared at a moderate price consistent with requirements in each case.

Trusting that I may have the pleasure of serving you soon, I remain,

Yours very truly,

———— Refracting Optician.

The foregoing interesting circular is being mailed to physicians in this city.

How long, O Lord, will the silent profession keep silence.

It seems not sufficient that we should learn wisdom from patent medicine venders, have the hard places in prescribing smoothed out by ready-made formulæ expansively labeled "Diarrhœa," "Hepatic," "Cough No. 1," but we must turn to the laymen for counsel and advice to clear away the cobwebs that festoon our domes of thought.

"There is a growing desire among the fraternity to know the exact state of the eye, while pursuing a course of medical treatment." How refreshingly *naive*, and yet how true! It is time indeed for the fraternity to pursue a course of medical treatment that the beam may be cast from its eye that it may see clearly to just what manner of fool it belongs.

Yet in the meantime, what an ideal state is hinted at in the circular. All men, laymen and physicians, laboring harmoniously together in the struggle against the common enemy. It approaches Plato's ideal republic. Send your gastritis patient to the dietary expert at the corner grocery when you have a growing desire to know the exact state of his stomach. Personally conduct him to the skilled operator attached to the meat market if you suspect that he has one appendix too many or that his brisket needs to be trimmed *a la mode.*

We would be cheerful touts for the butcher and baker specialists whom "higher medical education, accurate methods of diagnosis, and subdivision of labor" have spawned out upon the shamed face of the world.

It is time for the fraternity to look to that dignity of which we hear to much and see so little.

